# Impact of valvuloarterial impedance on left ventricular reverse remodeling after aortic valve neocuspidization

**DOI:** 10.1186/s13019-022-01760-7

**Published:** 2022-01-29

**Authors:** Naoki Yamamoto, Hisato Ito, Kentaro Inoue, Ayano Futsuki, Koji Hirano, Yu Shomura, Yasuhisa Ozu, Yoshihiko Katayama, Takuya Komada, Motoshi Takao

**Affiliations:** 1 Department of Thoracic and Cardiovascular Surgery, Mie University Hospital, 2-174 Edobashi, Tsu City, Mie 514-8507 Japan; 2 Department of Thoracic Surgery, Matsusaka Chuo General Hospital, 102, Kawai-Machi-Komou, Matsusaka City, Mie 515-8566 Japan

**Keywords:** Aortic valve neocuspidization, Aortic valve stenosis, Left ventricular geometry, Left ventricular reverse remodeling, Valvuloarterial impedance

## Abstract

**Background:**

Aortic valve neocuspidization (AVNeo) has emerged as a promising aortic valve procedure, and is expected to have a larger effective orifice area (EOA) than commercially available bioprostheses. It is, however, unclear which indices could facilitate left ventricular (LV) reverse remodeling after AVNeo. The aim of this study is to verify the impact of global left ventricular afterload on the LV reverse remodeling following AVNeo.

**Methods:**

Data-available consecutive 38 patients (median age, 77; interquartile range, 72.8–82.0) undergoing AVNeo for severe aortic stenosis were enrolled in this study. Preoperative and the last follow-up echocardiographic data were retrospectively analyzed including the valvuloarterial impedance (Zva), a marker of global LV afterload. Reduction in LV geometry index (LVGI) and relative wall thickness (RWT) were used as an indicator for LV reverse remodeling.

**Results:**

The Zva reduced in 24 patients (63.2%) during the follow-up period (median, 12 months). Reduction in Zva significantly correlated to improvement of LV geometry (LVGI (r = 0.400, *p* = 0.013) and RWT (r = 0.627, *p* < 0.001)), whereas increase in EOA index did not significantly correlate to LVGI (r = 0.009, *p* = 0.957), or RWT (r = 0.105, *p* = 0.529)). The reduction in Zva was the multivariate predictor of LV reverse remodeling.

**Conclusions:**

Low global LV afterload led to significant LV reverse remodeling even after AVNeo, which could achieve better valve performance than the conventional bioprostheses.

## Introduction

Left ventricular hypertrophy (LVH) is a well-recognized risk factor of left ventricular (LV) dysfunction independent of the severity of the valvular load in patients with severe aortic valve stenosis (AS) [1]. In addition to LVH, LV concentric remodeling which is represented either by an increased LV mass-to-volume ratio (left ventricular geometry index, LVGI), or by an increased LV wall thickness-to-internal diameter ratio (relative wall thickness, RWT), has been shown to independently predict LV dysfunction and adverse cardiovascular events [2–4].

Aortic valve replacement (AVR) could facilitate LV reverse remodeling with increased effective orifice area (EOA). Prosthesis-patient mismatch (PPM) after AVR, however, may have a negative impact on LV reverse remodeling and even on survival [5]. On the contrary, even if a patient has PPM, LV reverse remodeling could occur, and EOA alone could not fully explain the mechanism of LV reverse remodeling [6].

Valvuloarterial impedance (Zva) is an echo-derived measurement of global LV afterload including both valvular load and systemic arterial compliance (SAC) [7]. We have reported that low Zva facilitated LV reverse remodeling after AVR with externally wrapped bioprosthetic valves [8]. The aortic valve neocuspidization (AVNeo) has emerged as a promising aortic valve procedure, in which aortic valve cusps are reconstructed with glutaraldehyde-treated autologous pericardium [9], and is expected to have a larger EOA than commercially available bioprostheses [10]. It is hypothesized that low global LV afterload may also have a positive effect on LV reverse remodeling even after AVNeo. The purpose of this study is to verify the impact of global LV afterload on LV reverse remodeling following AVNeo for AS patients.

## Methods

### Study design

This retrospective observational study has been approved by the institutional review board of Mie University Hospital (approval date, April 11, 2019; approval number, H2019-056), and informed consent was obtained in an opt-out fashion. All data were retrieved from the medical records.

Data-available 38 patients (median age, 77; interquartile range (IQR), 72.8–82.0) undergoing AVNeo for severe AS between April 2013 and June 2018 were enrolled in this study. Patients undergoing emergency surgery, those with more than mild aortic insufficiency, those with atrial fibrillation, or those without follow-up data after operation were excluded from the study. The median follow-up period after surgery was 12 months (IQR, 6.0–31.5). This follow-up period was considered appropriate because previous studies have shown that maximum LV mass regression occurred within the first 3–4 months and that much smaller declines were observed over the subsequent years [11, 12].

### Surgical procedure

**Fig. 1:**
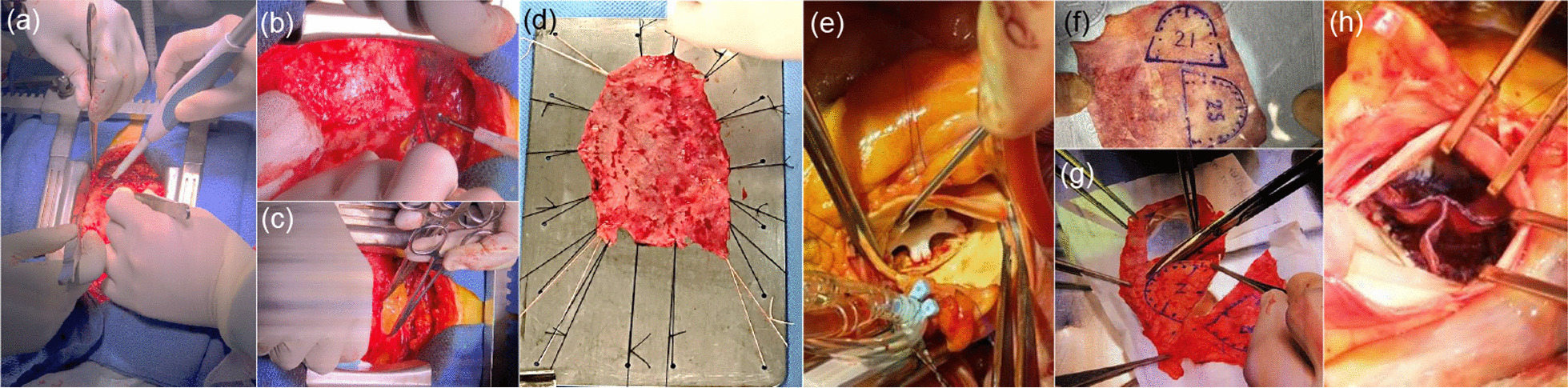
Surgical procedure of AVNeo. **a** Harvest of the autologous pericardium after median sternotomy. **b** and **c** Separation of the autologous pericardium. **d** Autologous pericardium being prepared with 0.6% glutaraldehyde solution. **e** Measurement of each annular distance between commissures with the original sizer system after removal of the leaflets. **f** Trimming of the autologous pericardium corresponding to the measured leaflet sizes using the original template. **g** Cutting out of the neo-valve cusps. **h** Reconstructed neo-aortic valve

The AVNeo was thoroughly explained elsewhere [9]. Briefly, after median sternotomy, the autologous pericardium is prepared with 0.6% glutaraldehyde solution for 10 min, and is rinsed for six minutes three times in saline solution. The distances between each commissures are measured with the original sizer system after removal of the aortic valve leaflets. The new cusps are trimmed from the treated autologous pericardium by using the original template. Finally, the annular margin of a pericardial cusp is attached to each annulus with 4-0 monofilament running sutures. The commissures are reinforced with additional pledgeted 4-0 mattress sutures (Fig. 1).

### Echocardiography analyses

Echocardiography analyses were performed according to the American Society of Echocardiography [13]. Serial transthoracic echocardiography were performed within three months preoperatively, and at the last follow-up (median, 12 months; IQR, 6–31) in all patients. LV volume was calculated with modified Simpson’s method, and LV ejection fraction (LVEF) was calculated as [(LV end-diastolic volume—LV end-systolic volume)/LV end-diastolic volume] × 100. LV mass was calculated using the formula recommended by the American Society of Echocardiography and indexed to body surface area (BSA) (left ventricular mass index (LVMI)) [14]. Concentric LV structural remodeling in patients with AS is considered as a compensatory response to LV load to reduce wall stress according to the law of La Place, and defined as LVGI > 1.5 [15, 16] or RWT > 0.42 [13]. LVGI and RWT were calculated as LVGI = LVMI / LV end-diastolic volume index [2, 15–17]; RWT = (interventricular septal wall thickness + posterior wall thickness (PWT)) / LV end-diastolic diameter (LVEDD) or 2 × PWT / LVEDD [13, 18]. Reduction in LVGI and RWT were used as an indicator for LV reverse remodeling representing less concentric geometry after AVNeo. LV stroke volume was calculated as LV outflow tract (LVOT) area × LVOT velocity–time integral (VTI), and indexed to BSA (stroke volume index, SVI). Early transmitral filling peak velocity (E) and transmitral atrial wave velocity (A) were measured with pulsed wave Doppler. The E/A ratio was used as an index of LV diastolic function. Early diastolic mitral annular velocity (E’) was measured and E/E’ ratio was calculated to estimate LV filling pressure. Doppler echocardiographic assessments of AS severity included peak transvalvular flow velocities, and mean transvalvular pressure gradient (TPG) calculated by the modified Bernoulli equation. EOA was calculated using the standard continuity equation with pulsed-wave VTI ratio between LVOT area and aortic valve area, and indexed for BSA (effective orifice area index (EOAI)): EOA (cm^2^) = π × (LVOT radius)^2^ × (LVOT VTI / aortic valve VTI). For more accurate estimation of aortic valve area, energy loss coefficient (ELCO) was calculated and indexed for BSA (energy loss index (ELI)) using the formula: ELI (cm^2^/m^2^) = [(EOA × Aortic area)/(Aortic area – EOA)]/BSA [19]. The stroke work loss was calculated using the formula: Stroke work loss (%) = [mean TPG/(systolic arterial pressure + mean TPG)] × 100 [20]. SAC was calculated as SAC (mL/m^2^/mmHg) = SVI/pulse pressure. Global LV afterload was calculated by the formula: Zva (mmHg/mL/m^2^) = (systolic arterial pressure + mean TPG)/SVI [7]. In addition, aortic valve dimensional changes at preoperative and at the last follow-up were analyzed on the parasternal long-axis view.

### Statistical analyses

Continuous variables were presented as median and IQR for non-normally distributed data, and dichotomous data were presented as numbers and percentages. Continuous variables were compared between preoperative and the last follow-up using the Wilcoxon signed-rank test. For evaluation of the correlations between global LV afterload or conventional aortic valve functional indices, and LVMI or left ventricular geometry, Pearson’s or Spearman’s correlation coefficient were calculated as appropriate. Multiple regression analysis was performed to identify independent factors for predicting LV reverse remodeling manifested by reduction in LVGI and RWT. The multivariate model included the change of Zva, EOAI, ELI, mean TPG and SAC between the values at the preoperative period and at the last follow-up. A *p* value less than 0.05 was considered statistically significant. All the statistical analyses were performed using IBM SPSS software, version 27.0 (IBM Corp, Armonk, NY).

## Results

### Patient profile

Preoperative patient characteristics are summarized in Table 1. Hypertension was present in 33 (86.8%) patients. Thirty (78.9%) patients had antihypertensive medications preoperatively. Seven patients underwent concomitant coronary artery bypass graft surgery, and three underwent concomitant procedures for the aorta.

**Table 1:** Preoperative patient characteristics

Variable	*N* = 38	
Age	77	(72.5–82.0)
Female (%)	20	(52.6)
Body surface area (m^2^)	1.50	(1.42–1.68)
HYHA functional classification		
I (%)	2	(5.3)
II (%)	14	(36.8)
III (%)	15	(39.5)
IV (%)	7	(18.4)
Hypertension (%)	33	(86.8)
Antihypertensive medications		
ACEI or ARB (%)	26	(68.4)
Beta-blocker (%)	4	(10.5)
Calcium channel blocker (%)	18	(47.4)
Diuretics (%)	9	(23.7)
Dyslipidemia (%)	22	(57.9)
Diabetes mellitus (%)	16	(42.1)
Hemodialysis (%)	3	(7.9)
Current smoker (%)	3	(7.9)
History of cardiac surgery (%)	0	(0.0)

Date are presented as median (interquartile range) or number (percentage)

*ACEI* angiotensin-converting enzyme inhibitor, *ARB* angiotensin II receptor blocker, *NYHA* New York Heart Association

### Operative and postoperative results

Two patients had bicuspid aortic valve and the others had tricuspid valve. The median circumferential distance of the aortic valve measured by the original sizer system was 75 mm (IQR, 71–85). The median operation, cardiopulmonary bypass and cardioplegic arrest times were 333 min (IQR, 307–407), 196 (IQR, 178–240) and 147 (IQR, 135–162), respectively. Thirty five (92.1%) patients had antihypertensive medications postoperatively. One patient needed reoperation due to a torn cusp; however, there were no infective endocarditis (IE), no thromboembolism, or no death and no other adverse cardiovascular events during the study period. Thirty five patients (92.1%) had improvement in postoperative New York Heart Association (NYHA) functional classification; however, four patients remained in NYHA II status. No patient were in more than NYHA II status.

### Echocardiographic and hemodynamic characteristics

Preoperative and the last follow-up echocardiography analyses are summarized in Table 2. Transvalvular peak velocity and mean TPG reduced significantly with increased EOAI and ELI. Stroke work loss also reduced after surgery. SAC had no improvement during the study period; however, Zva reduced in 24 patients (63.2%). Some patients showed little reduction of mTPG. These patients had relatively low preoperative mTPG, or low-flow, low-gradient AS due to low cardiac output. One patient had paradoxically higher Zva than the preoperative value because the patient had low-flow, low-gradient AS with preoperative LVEF of 43.5%, and the LVEF even decreased to 30.1% with reduced SVI postoperatively in spite of an improved mTPG from 42.4 mmHg to 5.5.

**Table 2:** Echocardiography results

	Pre-operation	Last follow-up	*p*
LAD (mm)	46.0 (41.7–50.0)	41.4 (38.3–44.3)	< 0.001
LVDd (mm)	50.2 (46.0–53.6)	45.4 (43.4–48.1)	< 0.001
LVDs (mm)	29.6 (26.8–36.2)	28.2 (26.2–29.6)	0.012
LVEDV (mL)	111.5 (97.3–139.7)	94.4 (85.1–108.1)	< 0.001
LVEDVI (mL/m^2^)	73.9 (62.8–91.2)	62.7 (54.9–73.3)	< 0.001
LVESV (mL)	34.2 (25.1–57.5)	29.8 (25.2–33.9)	0.012
IVST (mm)	12.4 (11.8–13.2)	11.0 (9.9–12.1)	< 0.001
PWD (mm)	11.8 (11.0–12.8)	10.5 (9.7–11.5)	< 0.001
RWT	0.49 (0.45–0.54)	0.46 (0.40–0.53)	0.060
LVMI (g/m^2^)	153.0 (127.3–187.7)	111.3 (96.8–135.5)	< 0.001
LVGI (g/mL)	2.06 (1.83–2.26)	0.68 (0.42–1.17)	< 0.001
SVI (mL/m^2^)	51.5 (45.8–58.0)	42.0 (36.2–46.7)	< 0.001
LVEF (%)	69.4 (61.9–75.3)	67.3 (62.3–72.7)	0.536
FS (%)	39.0 (33.4–44.1)	37.3 (33.2–41.4)	0.404
E/A ratio	0.70 (0.52–0.92)	0.88 (0.72–1.18)	0.007
E/E’	14.5 (12.2–19.0)	12.0 (9.5–19.0)	0.519
Aortic valve function			
Aortic regurgitation			< 0.001
None, n (%)	2 (5.3)	20 (52.6)	
Mild, n (%)	23 (60.5)	18 (47.4)	
Moderate, n (%)	13 (34.2)	0 (0.0)	
Severe, n (%)	0 (0.0)	0 (0.0)	
Max TPG (mmHg)	87.5 (68.4–104.2)	10.8 (8.3–15.9)	< 0.001
Mean TPG (mmHg)	52.8 (42.7–64.2)	5.9 (4.2–9.3)	< 0.001
Peak FV (m/s)	4.65 (4.10–5.13)	1.65 (1.40–2.00)	< 0.001
EOA (cm^2^)	0.82 (0.72–0.98)	2.15 (1.96–2.45)	< 0.001
EOAI (cm^2^/m^2^)	0.56 (0.45–0.63)	1.44 (1.31–1.62)	< 0.001
ELCO (cm^2^)	1.00 (0.83–1.22)	4.06 (3.38–5.11)	< 0.001
ELI (cm^2^/m^2^)	0.63 (0.53–0.79)	2.53 (2.19–3.48)	< 0.001
Zva (mmHg/mL/m^2^)	3.52 (3.07–4.29)	3.36 (2.86–4.24)	0.175
Stroke work loss (%)	28.7 (24.9–32.6)	4.44 (2.95–6.21)	< 0.001
SAP (mmHg)	129.5 (120.0–140.3)	135.0 (126.0–149.0)	0.338
MAP (mmHg)	89.0 (82.3–96.1)	92.0 (77.8–102.3)	0.546
SAC (mL/mmHg/m^2^)	0.79 (0.71–0.96)	0.61 (0.51–0.78)	< 0.001

Date are presented as median (interquartile range) or number (percentage)

*A* transmitral atrial wave velocity, *E* early transmitral filling peak velocity, *E’* early diastolic mitral annular velocity, *ELCO* energy loss coefficient, *ELI* energy loss index, *EOA* effective orifice area, *EOAI* effective orifice area index, *FS* fractional shortening, *FV* trans-aortic valve flow velocity, *IVST* interventricular septal thickness, *LAD* left atrial diameter, *LVDd and LVDs* diastolic and systolic left ventricular diameter, *LVEDV* left ventricular end-diastolic volume, *LVEDVI* left ventricular end-diastolic volume index, *LVESV* left ventricular end-systolic volume, *LVEF* left ventricular ejection fraction, *LVGI* left ventricular geometry index, *LVMI* left ventricular mass index, *MAP* mean arterial pressure, *TPG* transvalvular pressure gradient, *PWD* posterior wall diameter, *RWT* relative wall thickness, *SAC* systemic arterial compliance, *SAP* systolic arterial pressure, *SVI* stroke volume index, *Zva* valvuloarterial impedance

### Aortic valve dimensional changes

**Fig. 2:**
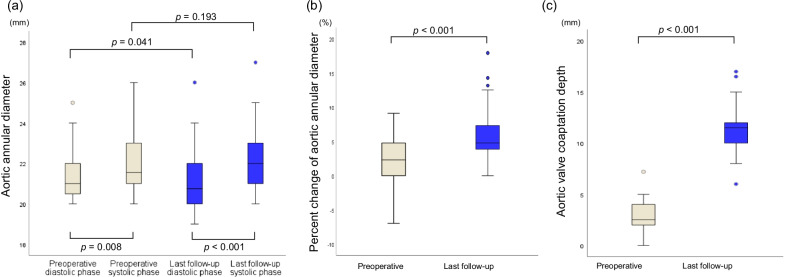
Aortic valve dimensional changes. **a** Changes in the aortic annular diameter during the cardiac cycle. **b** Percent change of the aortic annular diameter during the cardiac cycle at preoperative and at the last follow-up. **c** Change in the aortic valve coaptation depth

The aortic annular diameter at end-diastolic and end-systolic phase, and the aortic valve coaptation depth were analyzed in all cases by preoperative and the last follow-up echocardiography. Diastolic phase aortic annular diameter changed statistically significantly from 21.0 mm (IQR, 20.4–22.0) preoperatively to 20.8 (IQR, 20.0–22.0) at the last follow-up (*p* = 0.041). Systolic phase aortic annular diameter had no significant change between preoperative and the last follow-up (Fig. 2a). The aortic annular diameter after AVNeo changed significantly during the cardiac cycle from 20.8 mm (IQR, 20.0–22.0) in diastolic phase to 22.0 (IQR, 21.0–23.0) in systolic phase (*p* < 0.001), which was similar to the preoperative status. Percent change of the aortic annular diameter during the cardiac cycle increased significantly after AVNeo from 2.3% (IQR, 0.0–4.8) preoperatively to 4.8 (IQR, 3.8–7.3) (*p* < 0.001) (Fig. 2b). In addition, the aortic valve coaptation depth after AVNeo increased significantly from 2.5 mm (IQR, 2.0–4.0) preoperatively to 11.5 (IQR, 10.0–12.0) (*p* < 0.001) (Fig. 2c).

### LV reverse remodeling after AVNeo

**Fig. 3:**
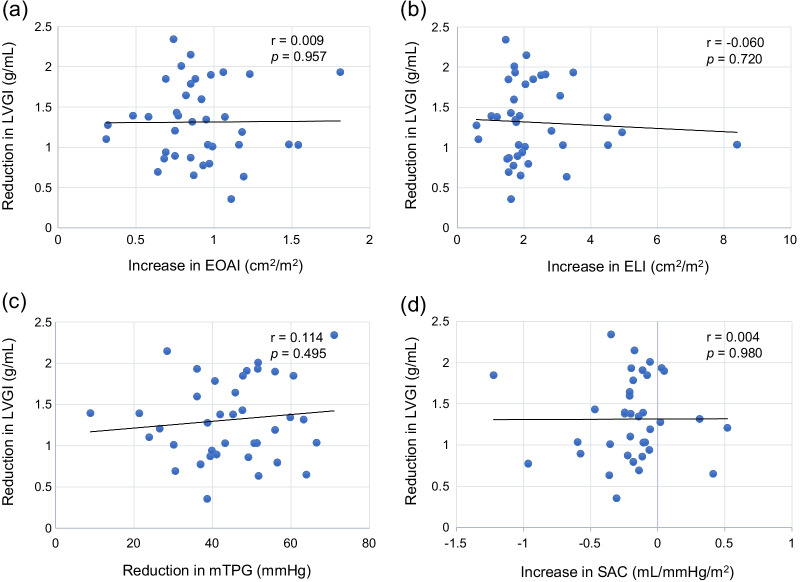
Correlation between reduction in LVGI and aortic valve functional indices, or SAC. **a** Correlation between reduction in LVGI and increase in EOAI. **b** Correlation between reduction in LVGI and increase in ELI. **c** Correlation between reduction in LVGI and reduction in mTPG. **d** Correlation between reduction in LVGI and increase in SAC

**Fig. 4:**
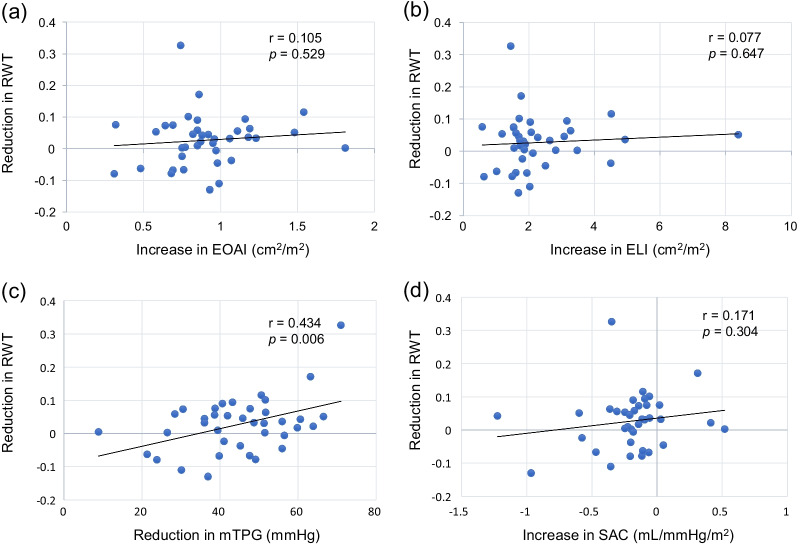
Correlation between reduction in RWT and aortic valve functional indices, or SAC. **a** Correlation between reduction in RWT and increase in EOAI. **b** Correlation between reduction in RWT and increase in ELI. **c** Correlation between reduction in RWT and reduction in mTPG. **d** Correlation between reduction in RWT and increase in SAC

**Fig. 5:**
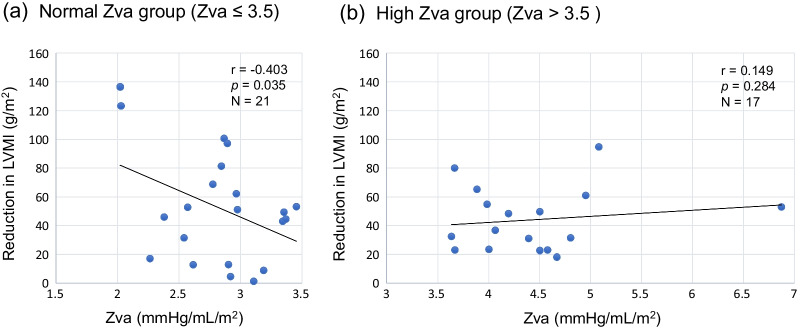
Correlation between Zva and reduction in LVMI. **a** Correlation between normal Zva group (Zva ≤ 3.5 mmHg/mL/m^2^) and reduction in LVMI. **b** Correlation between high Zva group (Zva > 3.5) and reduction in LVMI

**Fig. 6:**
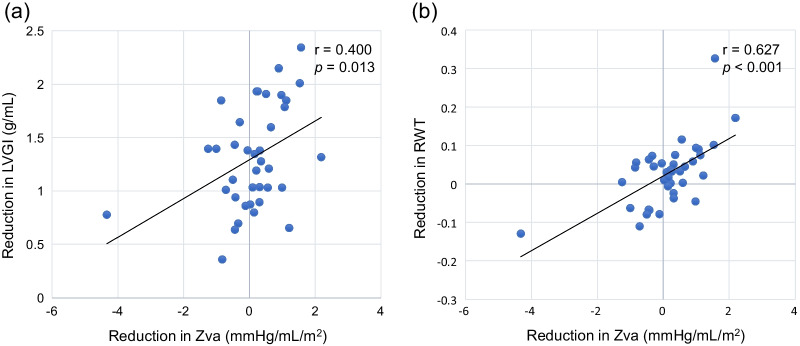
Correlation between reduction in Zva and changes in LV geometry. **a** Correlation between reduction in Zva and reduction in LVGI. **b** Correlation between reduction in Zva and reduction in RWT

LVMI reduced significantly from 153.0 g/m^2^ (IQR, 127.3–187.7) to 111.3 (IQR, 96.8–135.5) (*p* < 0.001). LVGI reduced significantly from 2.06 g/mL (IQR, 1.83–2.26) to 0.68 (IQR, 0.42–1.17) (*p* < 0.001), and RWT also reduced from 0.49 (IQR, 0.45–0.54) to 0.46 (IQR, 0.40–0.53) (*p* = 0.060). Correlations between changes of conventional aortic valve functional indices or SAC, and changes of LVGI or RWT are shown in Figs. 3 and 4. Reduction in mean TPG had significant correlation with reduction in RWT (r = 0.434, *p* = 0.006). Figure 5 shows correlations between postoperative Zva and reduction in LVMI. The normal Zva (≤ 3.5 mmHg/mL/m^2^) group had significant correlation with reduction in LVMI (n = 21, r = − 0.403, *p* = 0.035), whereas the high Zva (> 3.5) group did not. Figure 6 shows significant correlations between reduction in Zva and changes in LV geometry (reduction in LVGI (r = 0.400, *p* = 0.013) and reduction in RWT (r = 0.627, *p* < 0.001)). Reduction in Zva was the multivariate predictor of LV reverse remodeling, whereas changes of neo-valve functional indices and SAC were not (Table 3).

**Table 3:** Multivariate analyses for predicting left ventricular reverse remodeling

Variable	LVGI reduction			RWT reduction		
	β	95% CI	*p*	β	95% CI	*p*
Zva reduction	0.400	0.041–0.323	0.013	0.627	0.028–0.069	< 0.001
EOAI increase	− 0.021	–	0.892	0.059	–	0.659
mTPG reduction	− 0.068	–	0.694	0.205	–	0.156
ELI increase	− 0.103	–	0.512	0.012	–	0.929
SAC increase	− 0.363	–	0.055	− 0.315	–	0.050

*CI* confidence interval of non-standardized coefficients, *ELI* energy loss index, *EOAI* effective orifice area index, *LVGI* left ventricular geometry index, *RWT* relative wall thickness, *SAC* systemic arterial compliance, *mTPG* mean transvalvular pressure gradient, *Zva* valvuloarterial impedance

## Discussion

The major finding of the present study were that reduction in global LV afterload was a significant predictor of LV reverse remodeling after AVNeo for AS patients.

AVNeo with glutaraldehyde-treated autologous pericardium has emerged as a promising aortic valve procedure [9]. Several studies have reported good clinical results with AVNeo. AVNeo has been described to be a safe and reproducible procedure with excellent postoperative valve function and hemodynamics [21–25]. Among several studies with relatively long observation periods, Ozaki and colleagues [21] reported that actuarial freedom from death, cumulative incidence of reoperation, and the recurrent moderate or greater aortic regurgitation were 85.9%, 4.2%, and 7.3%, respectively in 850 patients with various aortic valve disease during the mean follow-up period of 53.7 ± 28.2 months. Freedom from death was comparable to that after AVR with bioprosthetic valves [22]. In addition, they also reported that among the 15 re-operated patients, 13 had IE, one had a broken suture material, and another had a cusp tear. In terms of valve function, they reported that postoperative echocardiography showed a peak TPG of 15.2 ± 6.3 mmHg at 8 years postoperatively. Other studies have shown that the patients undergoing AVNeo had less TPGs and larger EOA than those with conventional AVR, and that physiological motion of the aortic annulus was preserved after AVNeo [23]. Moreover, AVNeo has some advantages including large EOA in small aortic valve annulus, and anticoagulation-free postoperative management [10]. In the present study, trans-valvular flow velocity, TPGs, EOA and EOAI significantly improved, and LVMI reduced significantly after AVNeo. Mean TPG and EOAI after AVNeo in the present study were better than those with bioprosthetic AVR reported in other studies [24, 25]. In addition, the aortic annular motion was preserved even after AVNeo and the dynamic changes of annular diameter during the cardiac cycle increased more than the preoperative status. However, it has been unclear whether large EOA itself could solely contribute to LV reverse remodeling after AVNeo.

Conventional indices of neo-aortic valve function, such as trans-valvular flow velocity, TPGs and EOA, significantly improved after surgery in the present study. ELCO and ELI were also calculated for more accurate evaluation of the reconstructed valve. ELCO and ELI take into account the pressure recovery phenomenon, and is considered nearly equal to the valve EOA measured by catheterization [19]. In the present study, EOAI and ELI significantly increased after AVNeo; however, increase in EOAI or ELI were not correlated with LV reverse remodeling.

The Zva is an estimation of global LV afterload in AS patients [7], and can be measured by echocardiography in clinical practice. Consequently, the prognostic factors in AS patients such as blood pressure, arterial compliance and SVI as well as aortic valvular load are reflected in the formula of Zva. Aortic insufficiency and atrial fibrillation may affect TPGs and SVI, and lead to incorrect Zva quantification. In several studies, Zva was associated with improvement in LV function and in prognosis of AS patients [8, 26–28]. Hachicha and colleagues reported that survival was significantly lower in high Zva group (Zva > 3.5 mmHg/mL/m^2^) than normal (low) Zva group (Zva ≤ 3.5) or the age- and sex-matched control population [26]. Huded and colleagues reported that the baseline Zva in severe AS patients could predict the prognosis after AVR [27]. Katsanos and colleagues reported that the baseline Zva was an independent predictor of the mid-term mortality after transcatheter aortic valve implantation [28]. We also reported that the postoperative Zva was significantly related to LV mass regression after AVR using externally wrapped pericardial valves, and that patients with a normal postoperative Zva had significant reduction in LVMI [8]. These studies show the importance of Zva in predicting LV reverse remodeling and the survival in AS patients. In the present study, postoperative Zva was significantly correlated with reduction in LVMI in normal postoperative Zva group. Moreover reduction in Zva was also significantly related to improved LV concentric geometry after AVNeo. On the other hand, 14 patients had higher postoperative Zva, whereas all of them had significantly improved mTPG and larger EOA by AVNeo. Decreased SVI, and increased blood pressure (11 of 14 cases, 78.6%) most likely contributed to this result, and had a negative impact on the Zva values.

In evaluating LV reverse remodeling in AS patients, it is necessary to consider the presence of hypertension in addition to aortic valve function. Hypertension, as well as valve load, has been associated with negative LV reverse remodeling. Helder and colleagues reported that lower systolic blood pressure with beta-blockers and calcium-channel blockers was associated with LV mass regression after AVR using the Trifecta bioprosthetic valve [29]. Other meta-analysis have shown that angiotensin-converting enzyme inhibitors, angiotensin II receptor blockers, and calcium channel blocker contributed to significant LV mass reduction in comparison with beta-blocker [30]. AS is not an isolated valve disease and should be considered as a systemic atherosclerotic process involving valve stenosis and reduced SAC, which may be often overlooked in daily clinical practice. In the present study 92.1% of patients were on various antihypertensive medications at the last follow-up, and SAC significantly improved during the follow-up period. LV reverse remodeling after AVNeo may be facilitated if the global LV afterload is maintained as low as possible with proper use of vasodilators for the treatment of hypertension which often coexists in AS patients.

## Limitations

The main limitation is its retrospective nature in a single center with a small number of cases during a relatively short study period. The midterm outcomes after AVNeo are favorable; however, the longest follow-up period even in the longest series is 118 months [21], which is not considered long enough to ensure the durability of the neo-valve. On the other hand, the prosthetic valve replacement is still the gold standard treatment because of the good durability [31]. Therefore, it is difficult to draw any definite conclusions in comparison to the prosthetic valve replacement from the present study. The durability of the neo-valve needs to be validated by large-scale, long-term, multicenter and randomized prospective studies in the future, and then it may be possible to compare the functions of the neo-valve with that of prosthetic valves in detail.

## Conclusions

The reduction in Zva, an index of the global LV afterload, was significantly correlated to LV reverse remodeling after AVNeo. This might suggest that reducing the global LV afterload is important factor in enhancing LV reverse remodeling even after AVNeo which could make larger EOA than the usual bioprostheses. Zva could be used as a therapeutic target for LV reverse remodeling after surgery.

## Data Availability

The datasets used and/or analyzed during the current study are available from the corresponding author on reasonable request.

## Abbreviations

AVNeo: Aortic valve neocuspidization
EOA: Effective orifice area
LV: Left ventricular
Zva: Valvuloarterial impedance
LVGI: Left ventricular geometry index
RWT: Relative wall thickness
LVH: Left ventricular hypertrophy
AS: Aortic valve stenosis
AVR: Aortic valve replacement
PPM: Prosthesis-patient mismatch
SAC: Systemic arterial compliance
IQR: Interquartile range
LVEF: Left ventricular ejection fraction
BSA: Body surface area
LVMI: Left ventricular mass index
PWT: Posterior wall thickness
LVEDD: Left ventricular end-diastolic diameter
LVOT: Left ventricular outflow tract
VTI: Velocity–time integral
SVI: Stroke volume index
E: Early transmitral filling peak velocity
A: Transmitral atrial wave velocity
E’: Early diastolic mitral annular velocity
TPG: Transvalvular pressure gradient
EOAI: Effective orifice area index
ELCO: Energy loss coefficient
ELI: Energy loss index
IE: Infective endocarditis
NYHA: New York Heart Association
